# Exploring the associative learning capabilities of the segmented attractor network for lifelong learning

**DOI:** 10.3389/frai.2022.910407

**Published:** 2022-08-01

**Authors:** Alexander Jones, Rashmi Jha

**Affiliations:** Department of Electrical Engineering and Computer Science, University of Cincinnati, Cincinnati, OH, United States

**Keywords:** attractor network, associative memory, lifelong learning, associative data, learning techniques

## Abstract

This work explores the process of adapting the segmented attractor network to a lifelong learning setting. Taking inspirations from Hopfield networks and content-addressable memory, the segmented attractor network is a powerful tool for associative memory applications. The network's performance as an associative memory is analyzed using multiple metrics. In addition to the network's general hit rate, its capability to recall unique memories and their frequency is also evaluated with respect to time. Finally, additional learning techniques are implemented to enhance the network's recall capacity in the application of lifelong learning. These learning techniques are based on human cognitive functions such as memory consolidation, prediction, and forgetting.

## Introduction

Lifelong learning is a task that many living organisms must face every day. The world is highly dynamic and full of surprises, and it is up to the brain to decide how an organism should behave and take actions based on its current and past situations. Lifelong learning can be defined as the capability of a neural system to recall a wide range of information that can change over time (Biesialska et al., 2020). Since an organism's environment and situation can change, it must be able to constantly adapt and learn new things. This task presents two critical challenges, however (Sodhani et al., 2019). First, the organism's memory must not undergo the process of catastrophic forgetting (McCloskey and Cohen, 1989). In these cases, the system's recall capability suffers dramatic degradation in its recall capability while attempting to learn. The second issue is tied to the first and called capacity saturation (Parthipan, 2019). In these scenarios, the memory “fills up” and is unable to realistically store any more memories within itself. The system will attempt to continue to do so however, and the result is the system suddenly becoming overburdened with information and then entering a state of catastrophic forgetting (Sodhani et al., 2019).

With the high amount of complex information present in lifelong learning, processing of information can become resource-intensive and costly. One technique that developed in organisms to help simplify complex relations between concepts and ideas is associative memory. First demonstrated by Pavlov's famous experiment, the concept he called classical conditioning showed how associations between certain stimuli or events can trigger targeted responses (Pavlov and Gantt, 1928). Since then, the concept (associative memory) has blossomed into a wide field within the neural network community where these types of high-level ideas can be related (Krotov and Hopfield, 2016; Hu et al., 2017).

One of the most popular forms of associative memory are Hopfield Networks (Hopfield, 1982). These networks are a variant of an attractor network that utilizes an array of recurrently connected neurons to form memories that can recalled later. This approach to associative memory is not without its limitations, however. The calculated memory capacity of the network is in the realm of 12–15% of the its neuron count (Gardner, 1987). Other subsequent works also created neural networks with improved memory capacity for associative learning (Storkey, 1997; Davey and Hunt, 1999). In 2019, a network was defined called the segmented attractor network (SAN) that surpassed the memory capacity of these previously defined networks (Jones et al., 2019). The network is structured like a Hopfield network where processing is performed *via* recurrent connections but utilizes a discretized scheme for allocating information like content-addressable memory (Pagiamtzis and Sheikholeslami, 2006). By splitting information into separate categories called sets, the network connects and associates information across categories to form memories. These memories can then be recalled at later times by only using partial input in a process called pattern completion (Hunsaker and Kesner, 2013). The work showed how the network's hit rate changed with respect to various factors such as network size, number of memories in the network, etc.

In the case of the SAN, its current design allows the formation high-level associative memories, but in a limited fashion. If one were place it into a dynamic environment where it might encounter a large range of inputs over a period, its learning capabilities would be limited due to the simplistic Hebbian nature of the network's memory formation. To aid the SAN into becoming a network deployable to a lifelong learning environment, one can look to biology for inspiration.

Miraculously, many organisms can perform associative memory with ease in highly dynamic environments (Pontes et al., 2019). Humans can easily relate complicated ideas with one another, and organisms as simple as the *C. elegans* worm have demonstrated basic characteristics of memory association (Stein and Murphy, 2014). There are theories as to how associative memory is formed (Tannenbaum, 2009; Ozawa and Johansen, 2018), but the entire process is not known. Other learning mechanisms within the brain could be assisting with associative processes to create meaningful relationships in the brain's neural connections. Exploring cognitive concepts such as prediction (Agnati et al., 2017) or the brain's tendency to forget (Baddeley et al., 2019) to see how they could potentially affect or enhance the performance of the segmented attractor network to make it suitable for lifelong learning environments. The success of such a model could lead to it aiding the artificial intelligence field in developing neural networks that are less specialized and more generic in their capabilities.

Finally, to properly evaluate the SAN's capability of forming and handling associative memories in a lifelong setting, using a dataset filled with high-level information would be of great value. This work defines such a dataset that is used to evaluate the SAN's efficacy across multiple metrics. Then, it gives an overview on the network's design and how it is simulated. Next, analysis on a basic version of the network is conducted. Finally, more advanced learning techniques based on cognitive concepts that could help enhance the network's capability in lifelong learning are proposed, tested, and discussed.

## Materials and methods

### The EHoS dataset

To properly evaluate the SAN's performance as an associative memory for lifelong learning, a dataset was created that could properly demonstrate its capability to relate high level concepts to one another (Jones, 2020). The created dataset is called the European Heads of State (EHoS) dataset and is composed of 700 leaders throughout European history. Each leader within the dataset is considered a memory and possesses eight different features across eight categories (i.e., sets) of data. These categories are: first name, last name/title, century of rule, the state they ruled, the position they held (e.g., king), their dynasty/family/political party, their cause of death, and how many years they reigned.

Within each set of information in the dataset, a collection of features exists that uniquely describe every leader. However, many leaders might share common features with one another (e.g., many Roman emperors, many French leaders with the name “Louis,” etc.). These different relations lead to different sets possessing a range of unique features. To help compare sets, the “uniqueness” factor, or U-Factor, describes how many unique features exist within each set with respect to the size of the dataset. For a given set, the U-Factor, *U*_*i*_, can be defined as


(1) $$U_{i}=\frac{M_{i}}{I_{tot}}\;$$

where *M*_*i*_ represents the total number of unique features within the set, and *I*_*tot*_ describes the number of memories in the dataset (*I*_*tot*_=700 for the EHoS dataset). The resultant U-Factors for every set within the EHoS dataset can be found in Table 1. This factor will become important when analyzing how recalling sets of varying sizes affects the hit rate of the network.

**Table 1:** Unique features and U-factors for sets in the EHoS dataset.

**Set**	**First name**	**Last name/Title**	**Century**	**State**	**Position**	**Dynasty/party**	**Cause of death**	**Reign**
***M**_***i***_*	328	190	29	22	24	117	9	59
***U**_***i***_*	0.469	0.271	0.041	0.031	0.034	0.167	0.013	0.084

### Network design

The segmented attractor network is originally described in Jones et al. (2019). The network discretizes how high-level, categorical information can be organized in a neural network. It divides all information into separate sets, where each set represents a category of information (e.g., name, color, time, etc.). Within each set, information is further split into elements called features that describe specific values within each set (e.g., red, green, blue, etc. for color).

Each feature within the network is represented by a single neuron. Sets within the network are represented by the collection of neurons that represent their features. All sets are arranged in a single, recurrently connected layer. The synapses between all features in the network are not fully connected, however. Features within the same set do not possess synaptic connections to relate themselves to one another. This design ensures that relations are focused on information in other sets, and not on features within the same set. Also, unlike other attractors such as the continuous variety (Yan et al., 2019), the connections are adaptable instead of preset for a specific function since the goal of the network is to extract information from observations.

**Figure 1:**
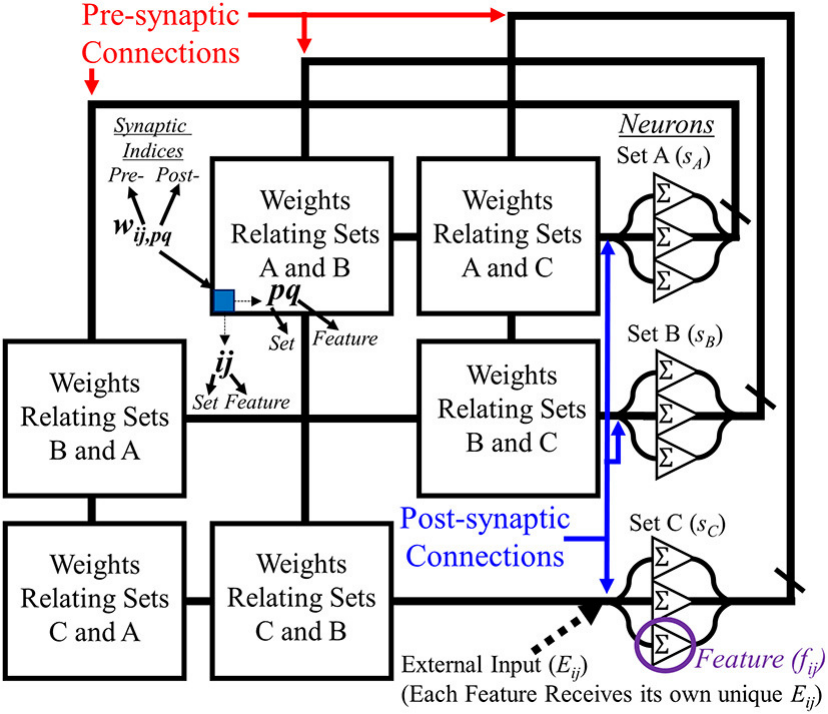
Diagram showing an example layout for the segmented attractor network. Recurrently connected neurons for post-synaptic (right) and pre-synaptic (top) connections along a synaptic grid that relates features from all sets with one another. Sets do not relate features within themselves with one another, forming an empty diagonal through the network to help keep information separate.

To properly utilize the SAN for the EHoS dataset, it is designed like the diagram in Figure 1. Each of the categories previously described are assigned as sets within the network, with each of the unique values within the sets for the dataset designated a neuron. These neurons are then recurrently connected to one another except for features within the same set.

At any given time step, *t*, in the SAN, the output of each neuron in the network is defined as


(2) $$f_{ij}\left(t\right)=E_{ij}\left(t\right)+\sum f_{ij}{\left(t\right)}^{*}\cdot w_{ij,pq}$$

where *E*_*ij*_*(t)* is the external input applied to each feature, *f*_*ij*_(t)^*^ is the recurrent output from neurons in the SAN, and *w*_*ij, pq*_ are the corresponding synaptic connections that recurrently connect those neuron signals to the specified feature. The *ij* and *pq* terms are the set/feature indices of the pre- and post-synaptic connections to the weight, respectively. In all other terms, *ij* serves as that terms' set/feature indices.

To place memories into the network from the EHoS dataset, external input is given to a collection of features in the network (one feature per set) to create a single memory. This memory will exist as a programmed combination of corresponding synaptic weights to the input. When external input is specifically supplied to a collection of features, the synapses within the grid that link those features to one another (i.e., *w*_*ij, pq*_) *via* their pre- and post-synaptic connections will be programmed from their original value (0) to the value, *v*_*ON*_ (*v*_*ON*_=0.001 for all cases shown in this work),


(3) $$w_{ij,pq}=\{\begin{array}{cc} w_{ij,pq},\; & f_{ij}<\left|E\right|\;or\;f_{pq}<\left|E\right|\\ v_{ON},\; & f_{ij}\geq \left|E\right|\;and\;f_{pq}\geq \left|E\right| \end{array}$$

In this work, *|E|* represents the magnitude of external input, which is defined as one. Due to how the values of *v*_*ON*_ and *|E|* are assigned, the recurrent feedback from the synaptic grid (i.e., Σ*f*${}_{ij}^{*}\cdot$*w*_*ij, pq*_) will never exceed *|E|*. This rule ensures external input is always trusted as correct.

To demonstrate how the SAN operates as an associative memory, the network can be shown to perform recall by applying external input to one feature in only half of sets within the network instead of all (i.e., four). This reduction in external input forces the network to perform pattern completion and use the associations previously made in its weights to complete an output response to recall memories it has previously observed. To determine the network's recalled state, the features with the maximum outputs from each set are defined as its final output, y.


(4) $$y=[\text{max}\left(s_{1}\right),\max\left(s_{2}\right),\ldots ,max(s_{n})]$$

In Equation (4), *n* denotes the number of sets within the SAN, and *s*_1_-*s*_*n*_ are labels for the sets of features in the network (*n*=8 for EHoS).

### Simulation process

**Figure 2:**
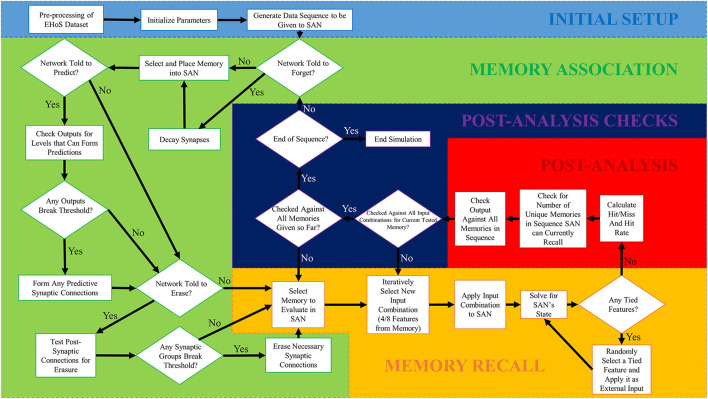
Simulation flowchart for the SAN simulation process conducted in MATLAB. There are five phases to the simulation process, with the first one only being ran once. The remaining four phases are ran iteratively in order to simulate the network over a total of 700 time steps. One memory from the EHoS dataset was introduced to the network at each individual time step, with the network's state being paused between time steps to evaluate its current recall capability.

To properly simulate the SAN on the EHoS dataset in a lifelong setting, a simulation framework was established in MATLAB to perform the entire process. This setup has several steps but can be split into five primary phases as shown in Figure 2. The first phase, initial setup, only occurs once during each simulation, and the final four phases occur repeatedly in sequence until the simulation is complete. The sequence uses no specialized MATLAB toolboxes.

#### Initial setup

The initial setup phase is a special one that only occurs once. First, the EHoS dataset's information is preprocessed. This process converts the text-based information into numerical data that can be more quickly analyzed by the code. If the dataset has not changed from the prior run, this step can be skipped.

The other two steps in the initial setup phase cannot be skipped. Next, the simulation's setup parameters such as *v*_*ON*_, additional learning capabilities to be used, etc. are established. The final initial step generates a sequence of memories from the EHoS dataset to be placed into the SAN. For the sake of comparison, every simulation demonstrated in this work utilizes the same randomly generated sequence of memories.

#### Memory association

During the memory association phase, the core step of introducing a new memory to the network is performed. The memory association phase will only introduce one memory to the network at a time per Equation (3). As an example, a memory from the EHoS dataset might be: Bourbon (dynasty) King (position) Louis (first name) XIV (title) of 17th century (primary century of rule) France (state), who ruled for 72 years (reign in years) and died of natural causes (cause of death). Each of these memories is selected at random from the EHoS dataset to be placed into the SAN (but never more than once). For a subsequent memory to be introduced into the SAN, the remaining three phases of simulation must be conducted first.

The remaining steps in the memory association phase relate to the additional learning capabilities that can be enabled within the SAN. Each of these steps can be seen in Figure 2 but will be discussed in greater detail in section 3.2.

#### Memory recall

The final three phases of the simulation occur in a cycled sequence that increases in length as the SAN simulation proceeds. Once this cycled sequence is over, the memory association phase can once again be conducted.

The first of these three phases, memory recall, iteratively selects memories from the part of the sequence shown to the SAN so far during memory association. It will then iteratively select half of the features from that memory and conceal them from the network. This concealment process forces the SAN to perform pattern completion to recall the remaining half of the memory it has been shown. The network will have its recall response analyzed for a winning feature within each set per Equation (4). If a winning feature cannot be found within a set after analysis, one of the tied features is randomly selected and has external input applied to its neuron (unless all outputs from a set are zero, then the set is ignored). The SAN is then reanalyzed for winning features. The process repeats until Equation (4) is solved.

#### Post-analysis

Post-analysis is a simple, record-keeping phase that tracks key performance metrics. These metrics include: hit rate with respect to the *U*_*avg*_ (i.e., average value of the four *U*_*i*_'s (Table 1) from the sets getting input during each recall trial) of the input provided to the SAN during memory recall, the number of unique memories that have been recalled at the current time step of the simulation, and how many times each memory from the EHoS dataset has been recalled at the current time step.

#### Post-analysis checks

The post-analysis checks phase is a quick, but key phase that determines when the simulation should either return to the memory association phase, memory recall phase, or finish the run. If the simulation has not finished recalling every possible combination of inputs from every memory already shown to the SAN up to the current time step, it will return the simulation to the memory recall phase. If the simulation has finished recalling every input combination of every memory up to the current time step, but the memory sequence still has more memories to introduce, the memory association phase will be initiated once more. If all memories have been properly recalled and all memories have been introduced to the SAN, the simulation ceases, and the run ends.

## Results

### Initial results

As a first demonstration of the SAN's functionality, a simulation is conducted that uses no special learning capabilities and uses the SAN's base behavior as described in section 2.2. Three metrics known as hit rate, the unique memory ratio, and recall occurrences are used to evaluate the network in a set of plots. They are described as follows:

#### Hit rate

**Figure 3:**
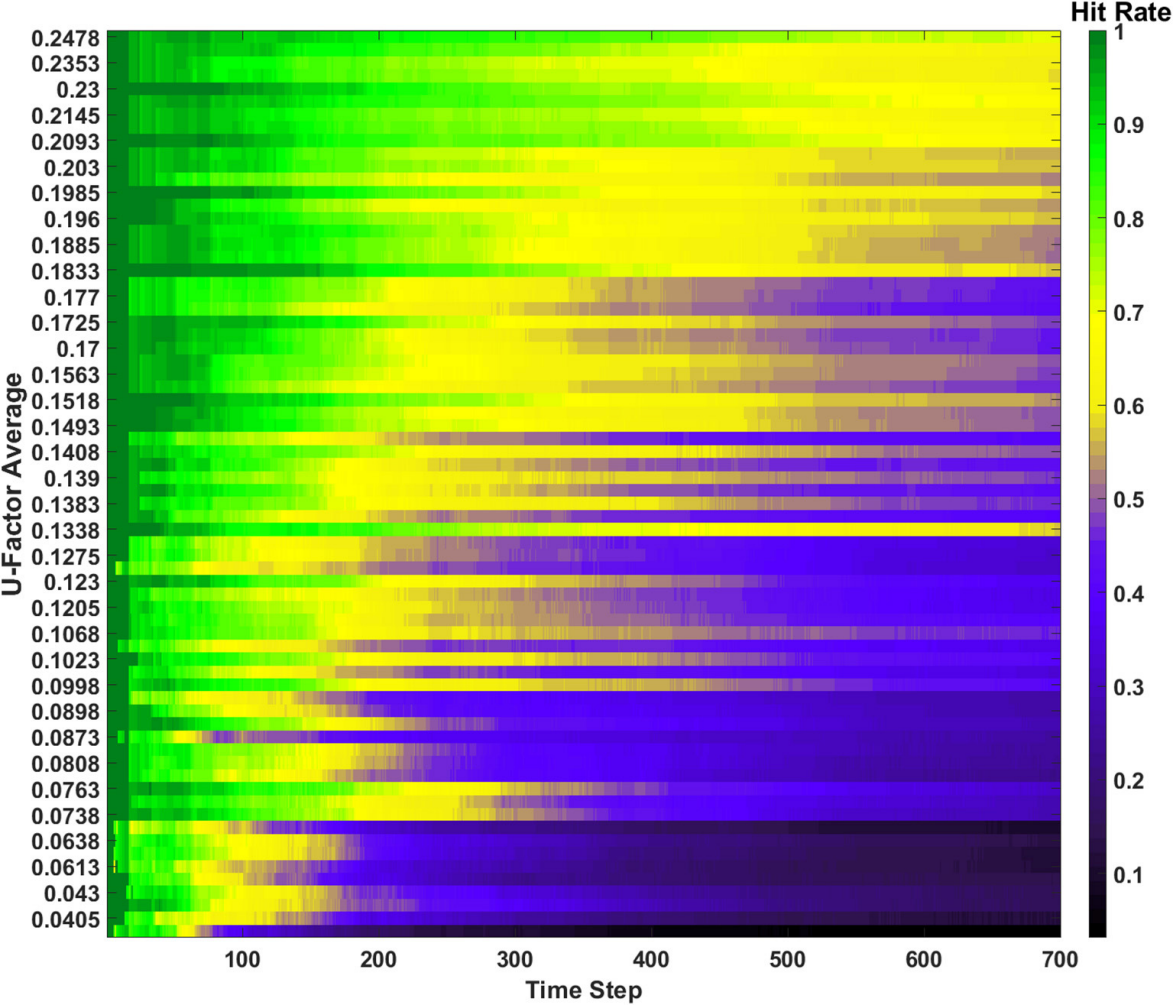
Hit rate for the baseline of the SAN against the EHoS dataset. The x-axis represents each time step throughout the simulation, while the y-axis represents the average U-Factor value (Table 1) for the four features shown to the network during each recall trial, *U*_*avg*_.

The hit rate of the SAN is defined as the number of times the network perfectly guesses a memory during the recall period. The hit rate for the SAN's baseline can be seen in Figure 3. The x-axis represents each individual time step, while the y-axis is the *U*_*avg*_ for the current combination of inputs being shown to the SAN during recall. Each new line on the y-axis represents a different combination of features from four different sets shown to the network. As the y-axis increases, so does the value of *U*_*avg*_, meaning the features shown to the network are coming from sets that are increasingly unique. If the SAN fails to guess the exact match of the full original memory previously placed into the network, the hit rate considers it a miss.

At each time step, one new memory is shown to the network. As time passes, memories are placed into the network and the hit rate overall decreases. This drop in performance is due to two factors. The first factor is that as more memories are placed into the network, its memory space becomes increasingly crowded; making the SAN more likely to recall an improper memory. The second factor is that the hit rate shown in Figure 3 is only based off memories it has seen so far. When *t* = 1, the SAN is only evaluated on the single memory is has observed so far. At *t* = 100, it is evaluated on those 100 memories it has been shown already, and so on.

The final observation to be made from Figure 3 is that it shows the hit rate across a two-dimensional plane instead of the usual single line with respect to time. Expanding the hit rate to also be shown with respect to *U*_*avg*_ was done to show how the type of information concealed from the network when performing recall has a large effect on how well the SAN performs. If a combination of inputs come from sets with a high *U*_*avg*_ value, the network is utilizing parts of the synaptic grid where information is more unique (i.e., less likely to be misconstrued). Other factors also affect the hit rate such as what memories have been shown to the network so far, but *U*_*avg*_ through the study revealed itself to be a primary controlling factor.

#### Unique memory ratio

**Figure 4:**
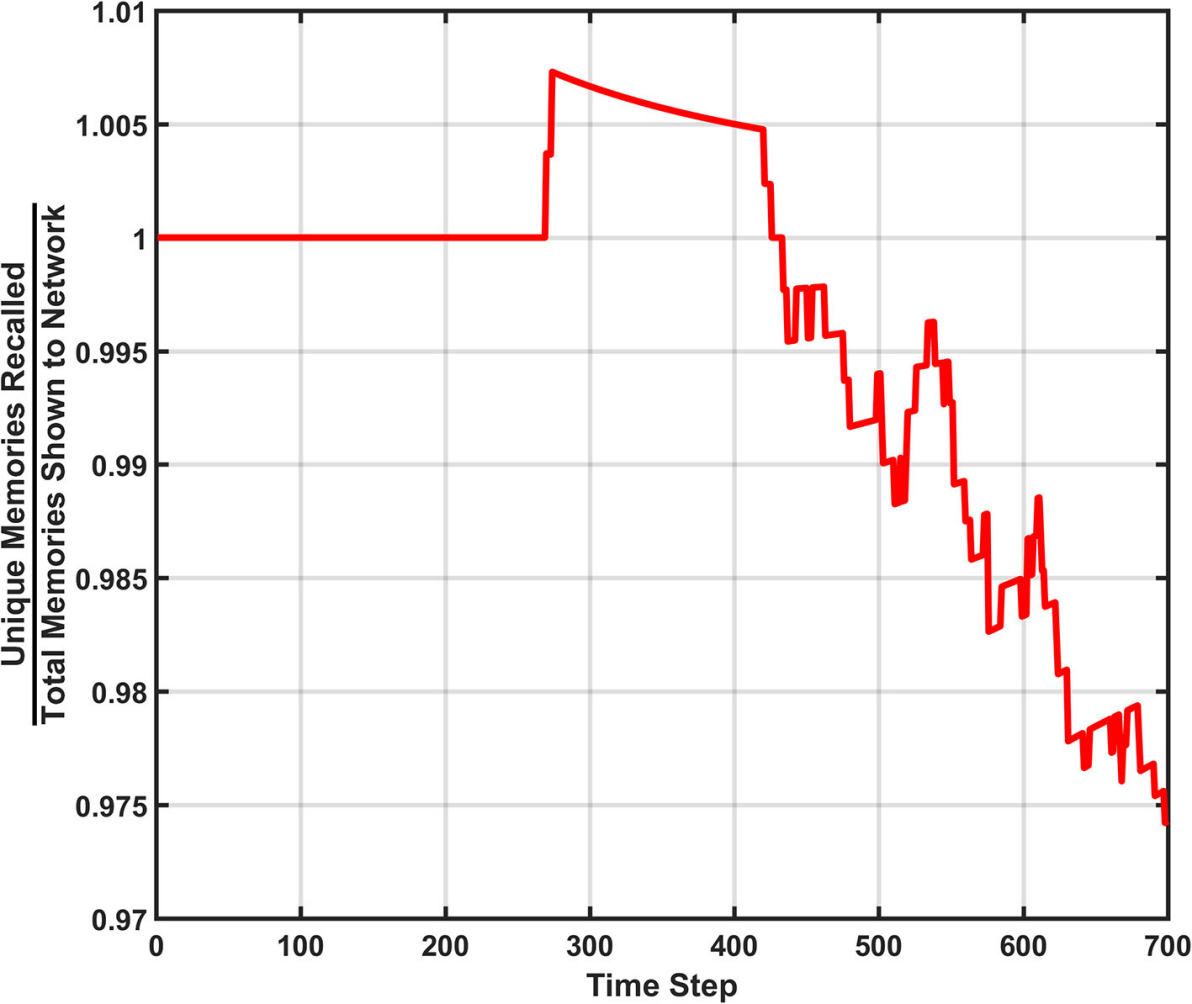
Unique memory ratio for the basic SAN run against the EHoS dataset. At each time step, the ratio tallies the amount of unique memories recalled in that time step's evaluation and divides it by the total number of memories shown to the network so far.

The second metric to monitor the performance of the SAN is called the unique memory ratio. The unique memory ratio of the SAN's baseline is shown in Figure 4 and shows how the ratio begins to decrease over time for the network. This ratio is not the same as hit rate, as it measures the number of unique memories recalled at every time step of the simulation. If a memory is shown to the network for recall purposes and the network fails to recall the remainder of the partially shown memory, hit rate considers that a miss. However, if the recalled memory is another memory that exists somewhere within the dataset, the unique memory ratio will count it toward its total.

As shown in Figure 4, the unique memory ratio ends at ~0.975, or 97.5%. This ratio means that after being shown all 700 memories, 97.5% of those memories were recallable under at least one of the input combinations tested when *t* = 700. It might not have been the original memory shown to the network during each recall trial, but the memory was still recallable under a certain condition. This value shows that even at its baseline, the SAN is a robust platform for associative memory.

One final feature of note within Figure 4 is the bump in the center of the figure. At this point, the unique memory ratio exceeds one. As previously stated, the unique memory ratio checks for any memories recalled that exist within the EHoS dataset, including ones not shown to the SAN yet. Between the 200th and 300th time steps the network begins to return recalled states of memories that the network has not been shown yet in addition to the ones already shown. This phenomenon occurs by statistical chance due to the SAN's synaptic states but does demonstrate a light form of prediction.

#### Recall occurrences

**Figure 5:**
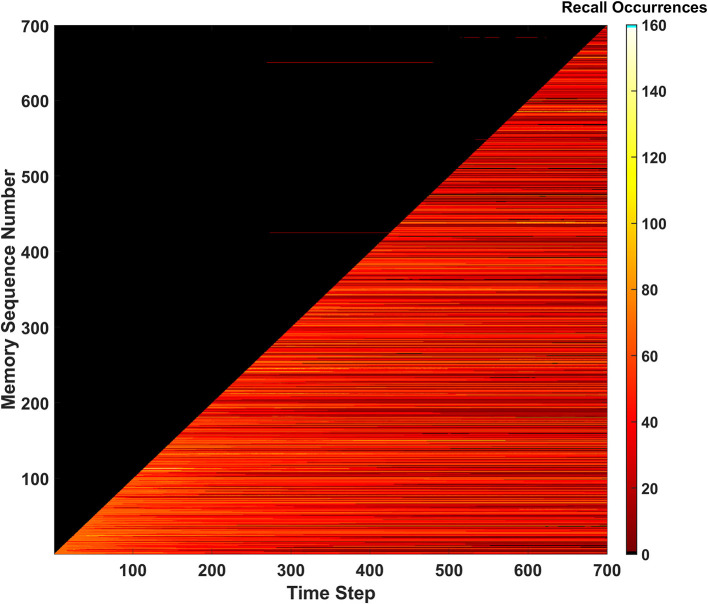
Recall occurrences heatmap for the basic SAN run against the EHoS dataset. This heatmap shows how many times at each time step a specific memory within the dataset was recalled. Memories are sorted along the y-axis with respect to the order in which they are shown to the network.

The third and final metric that monitors the SAN's performance is defined as recall occurrences. This metric is tracked *via* a heatmap as shown in Figure 5. The recall occurrences metric simply monitors how many times each memory within the EHoS dataset is recalled at every time step in the simulation. In the Figure 5 heatmap, the y-axis shows an indexed version of the memories per their order introduced to the SAN. This indexing creates a diagonal across the heatmap that demonstrates how the simulation introduces one new memory to the network per time step. Below the diagonal, a bright region of reds and yellows shows memories already placed into the network. Above the diagonal is a large, dark region that shows memories that have not been introduced to the SAN yet.

Within the dark region above the diagonal, a few anomalies appear as small, red streaks. These streaks are memories that have not yet been shown to the SAN but have been returned as recalled states at those specific time steps. This result again shows the light predictive behavior exhibited by the network that occurs by chance. These predicted memories appear and disappear as time passes and the SAN's internal state changes.

Another feature that occurs for a handful of memories is seen in the upper-right portion of the heatmap. As more memories are placed into the SAN and its grid becomes crowded, some memories begin to never be recallable even after being initially introduced. This phenomenon appears as black streaks that cut through the bright region beneath the diagonal to the final time step. These streaks do not occur often but appear to occur with increasing frequency as more memories are introduced. This observation alludes to the potential of datasets larger than EHoS having a larger problem of permanently unrecallable memories.

### Expanding the network's behavior

To address the shortfalls seen in the SAN's baseline and make it usable in a lifelong learning environment, additional learning behaviors could be introduced to the network to counter them. In addition, the previously observed prediction by chance behavior could also be further enhanced by modifying learning behavior in the network. Three extra learning rules for the network will be discussed and tested on the SAN to see how the network's behavior adjusts. Although these behaviors will not grant the SAN unlimited capacity, the goal is to help curate the critical memories it needs for its specified application.

#### Predictive behavior

**Figure 6:**
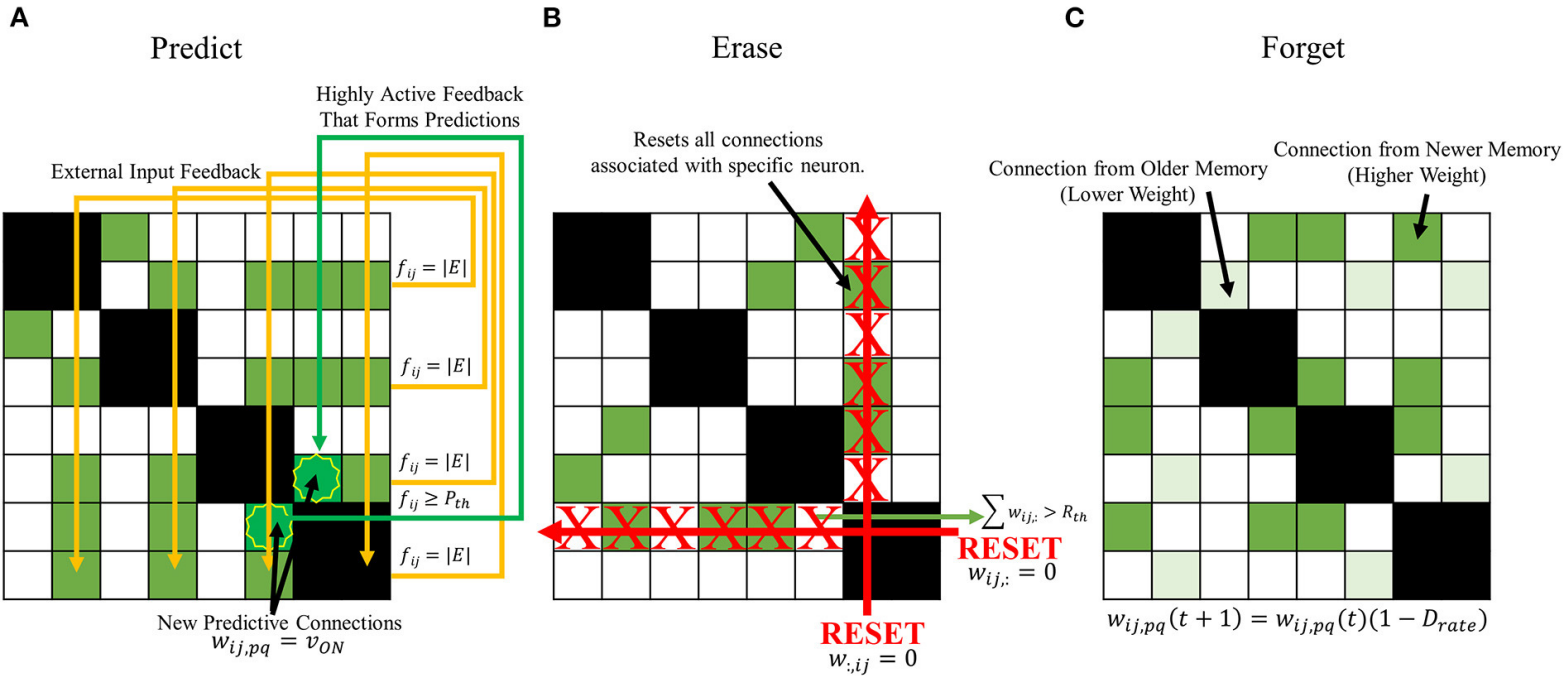
Proposed expanded learning behaviors to introduce to the SAN. **(A)** a predictive behavior that intends to enhance the network's already existing capability to randomly predict future concepts. If the output a neuron not receiving external input surpasses *P*_*th*_, it becomes associated with all other present external input. **(B)** an erase behavior that clears all synapses associated with a specific neuron if the number of programmed synapses surpasses *R*_*th*_. **(C)** a forgetting behavior that passively decays the value of a synapse's weight with respect to time according to Equation (7).

The first behavior to be introduced to the SAN is designed to more properly predict future memories the network might encounter. Seen in Figure 6A, the predictive behavior checks for neurons not receiving external input when a new memory is being introduced that have high output from synaptic grid feedback. A threshold is defined, *P*_*th*_, where if any neuron not currently receiving external input ever surpasses *P*_*th*_ during a memory association, it will associate itself with all other feature neurons currently being given external input. In other words,


(5) $$w_{ij,pq}=\{\begin{array}{cc} w_{ij,pq},\; & f_{pq}<P_{th}\\ v_{ON},\; & f_{pq}\geq P_{th} \end{array}$$

The primary objective of this behavior is to potentially predict future observations that might be witnessed by the SAN due to high correlation between features. This type of behavior is based on cognitive behavior seen in humans such as speculation or predicting future events based on the past (Agnati et al., 2017).

#### Erase behavior

One of the issues previously seen in the baseline of the SAN was the eventual degradation of the network's unique memory ratio as more memories were introduced. If the EHoS dataset was larger than 700 memories, this effect could be further exaggerated as the unique memory ratio continues to decay as hundreds or thousands more memories fill the synaptic grid. Extremely common features within a dataset can connect many memories' features to one another over time, and potentially cause the network's response to become less accurate. To combat this problem, another threshold is defined for the network, *R*_*th*_. If any neuron within the network ever has the sum of its pre-synaptic weights surpass *R*_*th*_, it is deemed a “commonly recurring feature,” and has its pre- and post-synaptic weights all reset to 0. These weights are not permanently removed, as in pruning, but instead simply reset. This behavior allows for these connections to once again be made in the future, if necessary, to aid in lifelong learning.

Shown in Figure 6B, the erase behavior is described by


(6) $$[w_{ij,:},w_{:,ij}]=\;\{\begin{array}{cc} {}[w_{ij,:},w_{:,ij}],\; & \sum_{}^{}w_{ij,:}<R_{th}\\ {}[0,0],\; & \sum_{}^{}w_{ij,:}\geq R_{th} \end{array}$$

where *w*_*ij*, :_ and *w*_:, *ij*_ each specify a column or row of weights, respectively. This learning mechanism aims to keep memories “more unique” in the network by removing commonly recurring features to make space in the synaptic grid for more memories in the future. This behavior is grounded in similar behavior seen in human brains in the field of neuroscience such as the sleep wake cycle (Zhou et al., 2011). When the brain enters its sleep state, it performs a memory consolidation process between the hippocampus and the neocortex (Lambert et al., 2020). Commonly observed, but unimportant information is removed and discarded in the process. This process allows for the brain to adapt to new information it sees daily.

#### Forgetting behavior

The third and final behavior that will be used on the SAN is one that aims to tackle the second problem seen in the network's baseline; permanently unrecallable memories. Some memories during the baseline appear to be unrecallable for the entire simulation run, even at the time step they are introduced to the network. This issue could be due to many factors, but primarily attributed to overcrowded areas of the synaptic grid where other memories already reside. A way to solve this problem would be a behavior that keeps the grid generally free as time passes. The erase behavior prevents select areas of the synaptic grid from saturating, but that might not be enough to combat this issue.

The most basic method that could be designed to combat this issue would be to introduce a general forgetting behavior to the network. Every synaptic connection would have its weight slowly decay over time. With this behavior demonstrated in Figure 6C, an equation can describe the weight of any synapse at the next time step as


(7) $$w_{ij,pq}(t+1)=w_{ij,pq}\left(t\right)\text{(}1-D_{rate})$$

where *D*_*rate*_ is the rate of decay for the synapse between time steps. Once again, this learning behavior is one based off human cognition. Naturally, as time passes, memories from the past become increasingly difficult to recall (Baddeley et al., 2019). Some notable memories might stand out, but many are forgotten.

### Expanded results

#### Predictive behavior

To test the first learning behavior for the SAN, the predictive behavior was used with *P*_*th*_=6*v*_*ON*_. The value defined for *P*_*th*_ means that if six or more pre-synaptic connections to a neuron receive external input from their pre-synaptic neurons, that neuron will associate itself with all other features currently being directly observed by the network.

**Figure 7:**
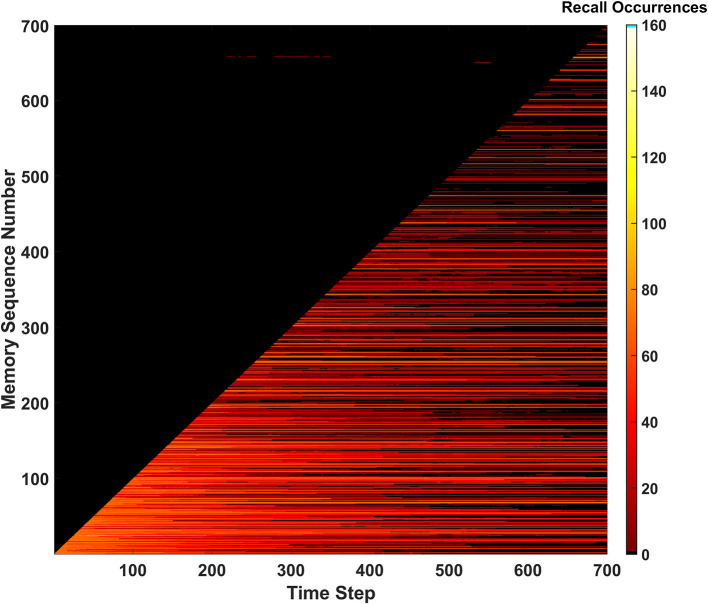
Recall occurrences heatmap for the predictive behavior when *P*_*th*_=6*v*_*ON*_. An increased amount of successful predictions were not seen, and the frequency of permanently unrecallable memories sharply increases as the simulation approaches its end due to the synaptic grid becoming saturated.

During the length of the simulation, a total of 527 predictions were made by the SAN. Seeing as 700 memories exist within the EHoS dataset, 527 is an incredibly high value. Due to the overactive predictive behavior, all metrics for measuring the SAN's success heavily dropped. One of the clearest indicators of this reduction in performance is recall occurrences shown in Figure 7. The hit rate and unique memory ratio are not shown here (Supplementary Section 1) but tell a similar story. Due to so many predictions being made, the synaptic grid quickly saturated; making it difficult to recall memories. In addition, the number of predictions made by the network in the dark region of Figure 7 did not increase. Another simulation was conducted at *P*_*th*_=7*v*_*ON*_, that had fewer predictions made (250), and did not differ much from the baseline of the SAN. This result indicated the behavior did not affect the network's performance.

A possible explanation behind the weak performance of the predictive behavior could be the EHoS dataset's nature. The average Hamming distance between memories in the dataset is 7.19 (e.g., maximum value is 8). This value means that memories are incredibly unique with respect to one another and only likely to share ~1 feature with one another. If the average distance was lower, the predictive behavior might have less of a negative impact on the SAN's performance while also increasing the amount of predictions made. This hypothesis means that the predictive behavior might be more useful in other, more heavily correlated datasets.

#### Erase behavior

**Figure 8:**
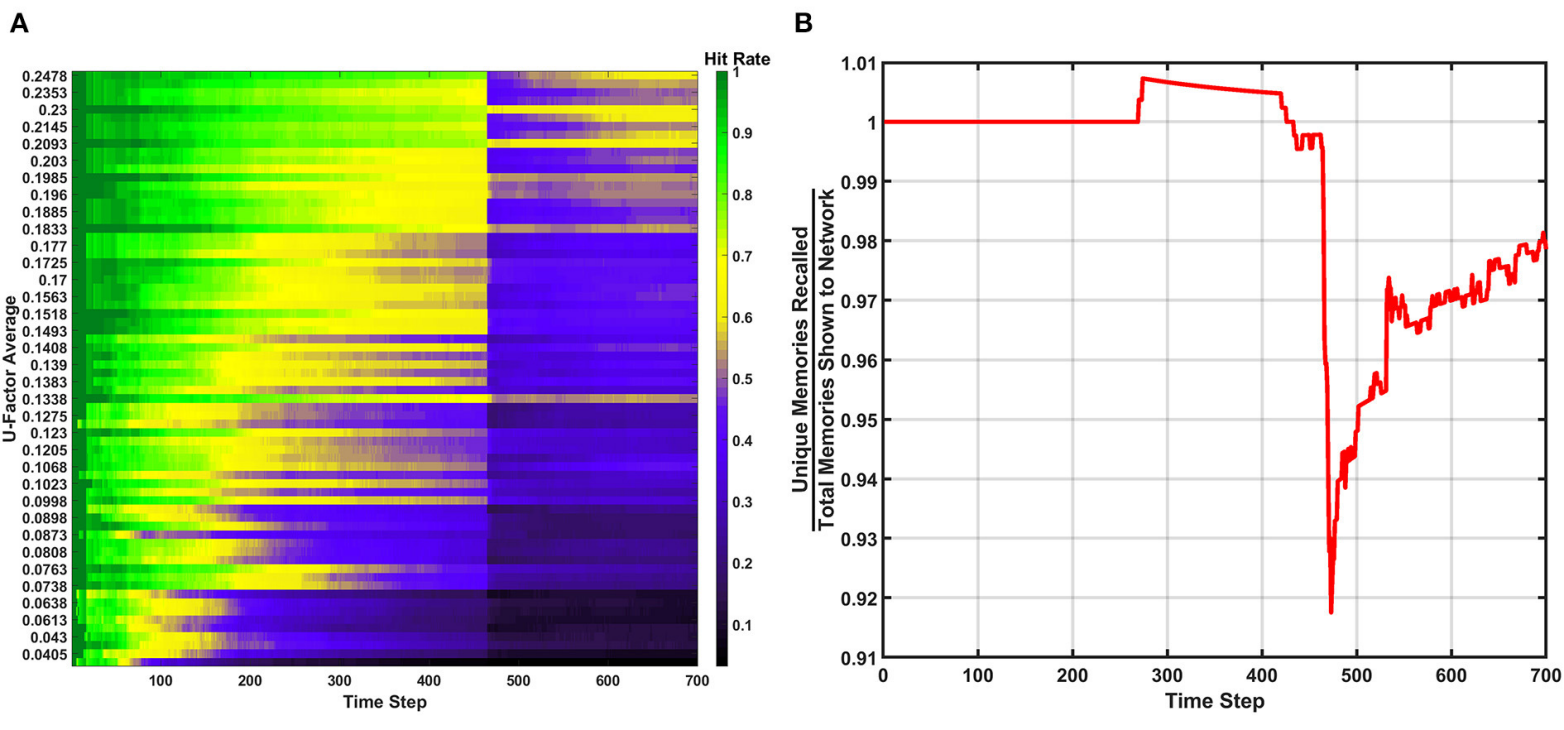
Results of the erase behavior when *R*_*th*_=400*v*_*ON*_. **(A)** The hit rate plot shows a clear drop when the erasure event occurred between the 400th and 500th time step. A period of post-erase recovery then ensues. **(B)** The unique memory ratio tells a similar story to the hit rate, where the erasure event incurs a short-term performance hit, but shows a post-erase recovery phase where the network recovers to a point slightly higher than the end result from the basic run.

The next learning behavior studied in the SAN aimed to generally increase long term performance within the network. The erase behavior's hit rate and unique memory ratio are shown in Figure 8 when *R*_*th*_=400*v*_*ON*_. The value defined for *R*_*th*_ means if 400 programmed synapses are pre-synaptically connected to the same neuron, that neuron will have all its pre- and post-synaptic connections reset. Due to this high threshold, only a single erasure event occurs during the entire simulation. This event can be clearly seen in Figure 8 where the fracture in the hit rate and the sudden decrease in the unique memory ratio both appear. What is seen after this erasure event is post-erase recovery. Since memories continue to be shown to the network after the erasure event, the hit rate and unique memory ratio both begin to increase again. The most impressive observation is that once the simulation has ended, the unique memory ratio has ended at ~98%. This result is ~0.5% higher than the value seen in the baseline, which demonstrates the erasure behavior being valuable for lifelong associative memory use.

**Figure 9:**
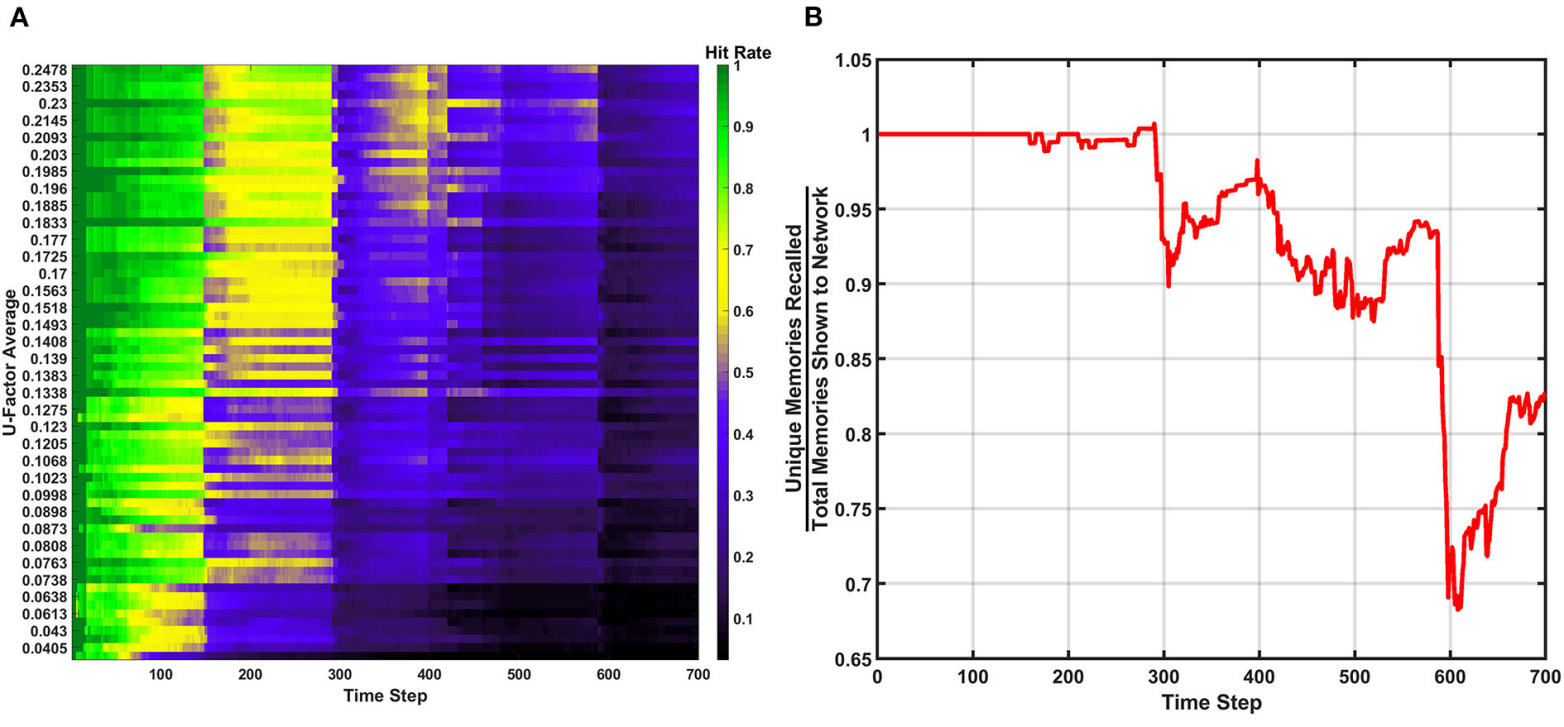
Results of the erase behavior when *R*_*th*_=200*v*_*ON*_. **(A)** The hit rate takes multiple hits from successive erasure events through the entire simulation. Phases of post-erase recovery do appear but are erased over time due to the high amount of erasure events. **(B)** The unique memory ratio shows multiple drops as erasure events occur. The ratio attempts to recover multiple times, but often fails to do so due to subsequent erasure events happening at a rapid pace.

If the value of *R*_*th*_ is decreased to a lower value (e.g., 200*v*_*ON*_), an example of a SAN that begins to underperform is revealed. In Figure 9 the hit rate and unique memory ratio for the more active erase behavior can be seen, where a couple dozen erasure events occur over the course of the simulation. The resulting performance shows that overuse of the erase behavior results in a network that is never able to fully experience post-erase recovery. This result when compared to Figure 8 demonstrates that rare use of the erase behavior can be very beneficial to long term network performance, but overuse is not advised.

#### Forgetting behavior

**Figure 10:**
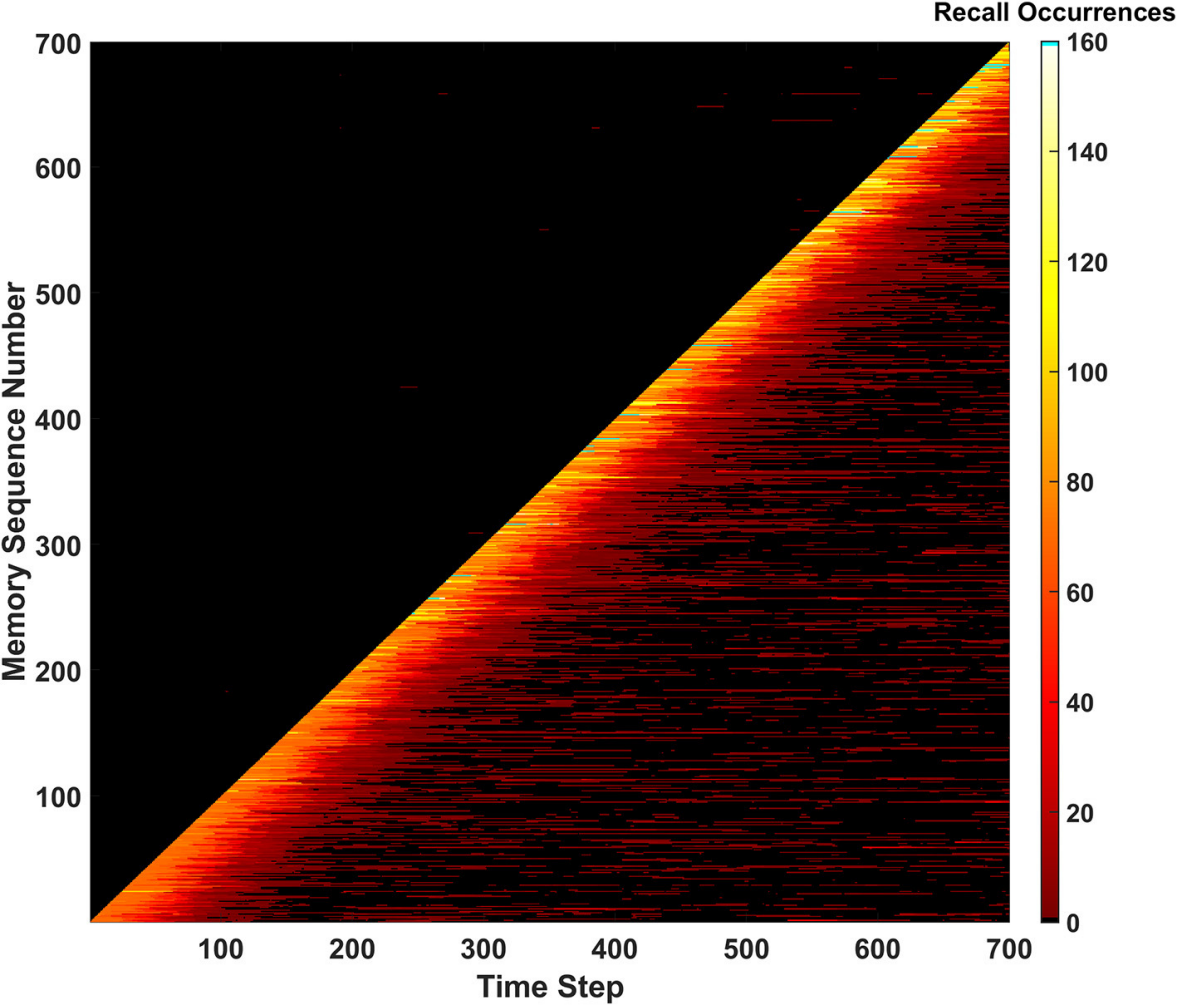
Recall occurrences heatmap for the forgetting behavior. Memories can be seen being forgotten as time progresses, and then remembered during intermittent periods for the remainder of the simulation (due to similar memories to the ones forgotten being newly introduced to the network).

The Forgetting behavior was tested in a simulation of the SAN with *D*_*rate*_=0.015 (i.e., 1.5%). Since the network is being told to forget memories as time passes, the hit rate and unique memory ratio metrics will suffer (Supplementary Section 3). The key issue the behavior is attempting to solve is permanently unrecallable memories. As shown in Figure 10, there are no longer any permanently unrecallable memories in the network, as no black streaks appear through the diagonal. This behavior could be useful in lifelong learning similar to the erase behavior where the memory is being curated by a heuristic over time. This behavior introduces a trade-off into the network, however. Implementing the forgetting behavior does ensure all new information is comprehended by network, but at the cost of forgetting memories as time passes. This parameter should be tuned for the network's environment. If the network exists within a constantly changing environment, having a higher *D*_*rate*_ value to ensure all new information is consistently stored in the network might be ideal, as older info will not be as relevant. In a more static environment, it might be best to have a much lower value of *D*_*rate*_, or none if completely static.

One other interesting behavior emerges in Figure 10. A “forgetting shelf” feature appears beyond the diagonal of the heatmap. After this shelf, memories become much less likely to be recalled due to *D*_*rate*_. However, many memories appear to temporarily reappear in certain places and then disappear once more. The explanation for this behavior is once past the forgetting shelf, a memory has not completely disappeared, but instead is weak enough to not be recalled. These older, weaker memories can later be reinforced by memories like them. This reinforcement causes the memory to be recallable again before the newer connections also decay. This type of phenomena could be defined as a long-term version of memory retrieval where specific cues are required known as ecphory (Frankland et al., 2019).

#### Behavior ensemble

**Figure 11:**
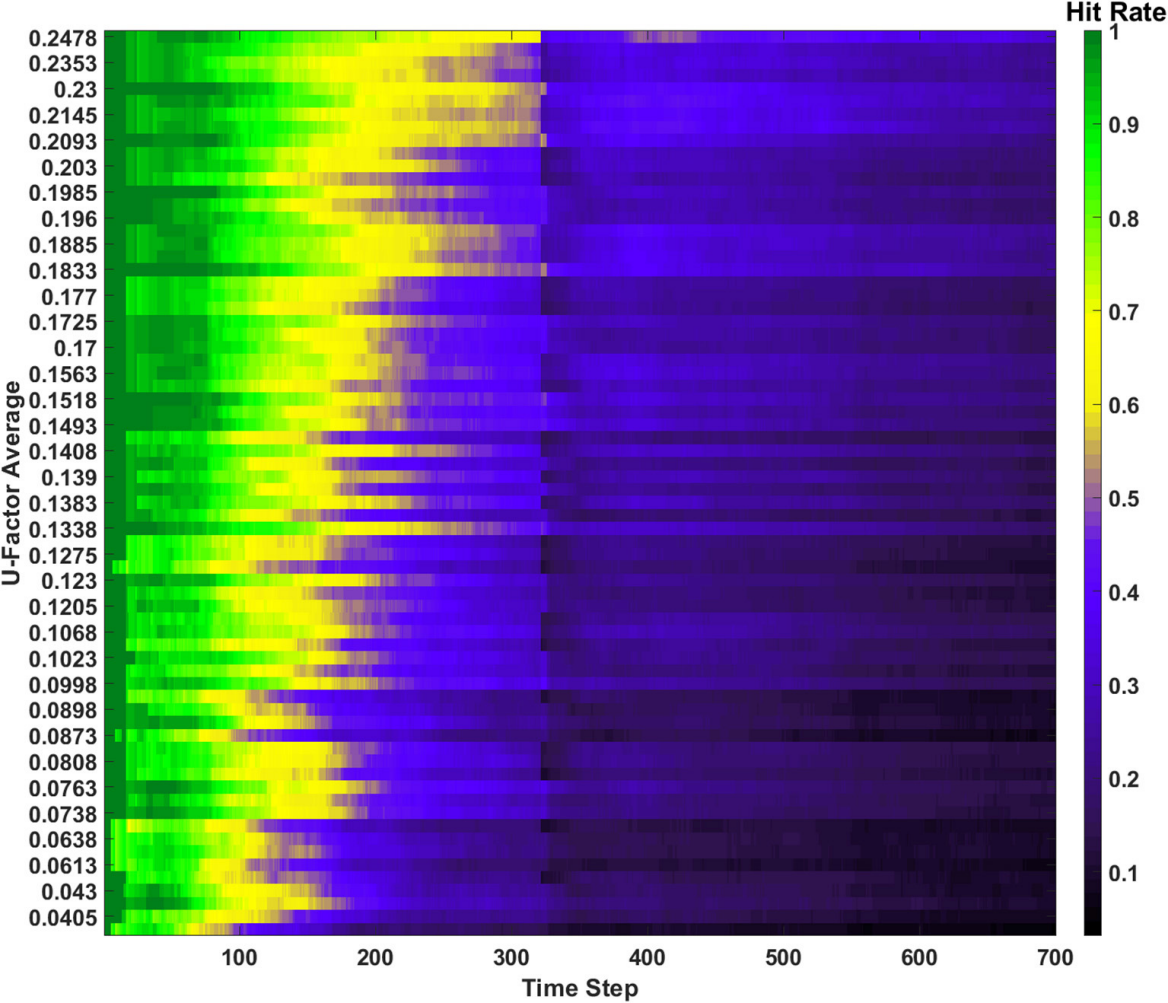
Hit rate for the behavior ensemble simulation of the SAN. One erasure event occurs, after which the post erase recovery is removed due to the forgetting behavior.

**Figure 12:**
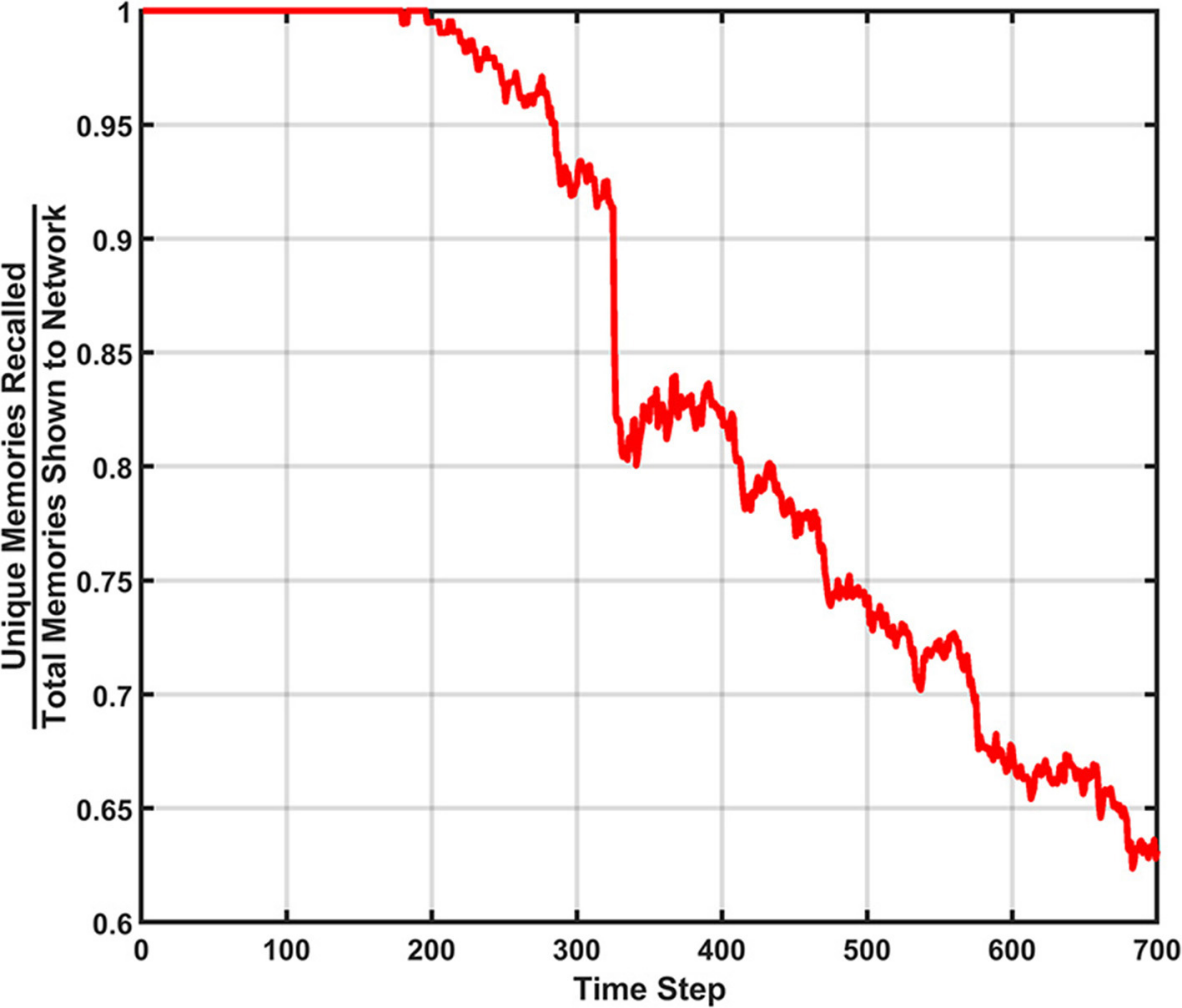
Unique memory ratio for the behavior ensemble simulation of the SAN. The erasure event can be seen in the steep drop in the ratio. The decay rate's effect can also be seen in the degradation of the ratio over time.

**Figure 13:**
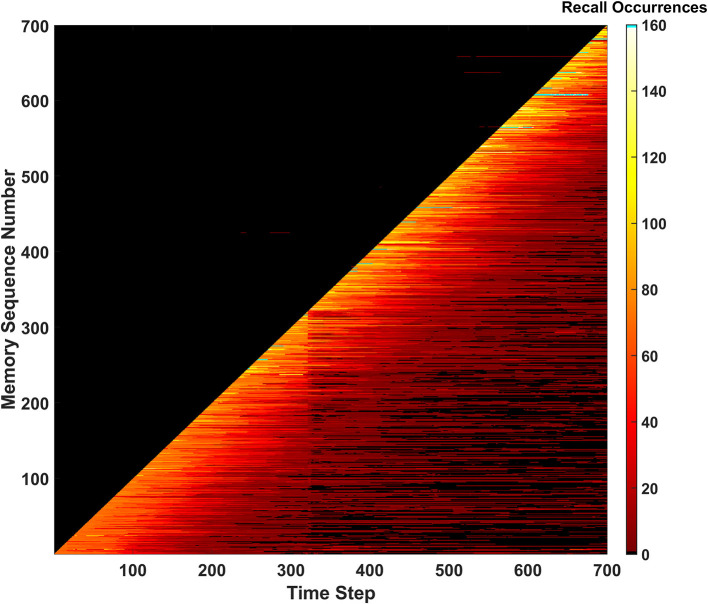
Recall occurrences heatmap for the behavior ensemble simulation of the SAN. Signatures of both the erase and forgetting behaviors can be seen in the forgetting shelf and fracture in the heatmap shortly after the 300th time step. All predictions above the diagonal were determined to be not made by the predictive behavior.

Now that all three behaviors have been individually studied, it is worth exploring the potential of all three learning behaviors being used together. The results of this demonstration can be found in Figures 11–13. For this simulation, *P*_*th*_=6*v*_*ON*_, *R*_*th*_=200*v*_*ON*_, and *D*_*rate*_=0.005 (i.e., 0.5%). During this simulation one erasure event occurred which is seen in all three metrics. The forgetting behavior can also be seen with the decrease in hit rate (removing post-erase recovery in Figure 11), unique memory ratio, and the (less prominent) forgetting shelf in Figure 13. The predictive behavior attempted to make many predictions, but they were again all from chance (Supplementary Section 4). The results of the ensemble show that behaviors can be combined to increase the network's behavioral complexity.

## Discussion

The results of all the expanded behaviors including the ensemble is quickly summarized in Table 2. Overall, the results from the SAN and its expanded behaviors show promise in its potentials for lifelong learning. The network's introduced learning rules help it solve some of the critical problems in lifelong learning such as catastrophic forgetting and memory saturation (Parisi et al., 2019). Although the network does forget memories over time, the learning rules introduced ensure that the information being forgotten is less critical since it's either extremely common (handled by erase behavior) or extremely rare (handled by forgetting behavior).

**Table 2:** Summary of expanded behavior results.

**Behavior**	***P_*th*_*(*v_*ON*_*)**	***R_*th*_*(*v_*ON*_*)**	***D_*rate*_***	**Result**
Predict	6	*-*	*-*	No prediction increase. Predictions were by chance.
	7	*-*	*-*	No prediction increase. Predictions were by chance.
Erase	*-*	400	*-*	UMR increased. One erasure event occurred.
	*-*	200	*-*	No UMR increase. Too many erasure events.
Forget	*-*	*-*	0.015	No permanently unrecallable memories. Memories fade with time.
Ensemble	6	200	0.005	All previous behavior signatures observed.

Other studies (Chaudhry et al., 2019) have shown where other types of networks have been benchmarked against more standard datasets such as MNIST or CIFAR. Similar results were shown in those demonstrations using a type of forgetting behavior where the network would slowly forget older tasks over time. Additionally, results in the accuracy from the models in Chaudhry et al. (2019), when compared to the hit rates results from this work show similar results. When ran against the CIFAR dataset, the best models from Chaudhry et al. (2017), capped at ~60% accuracy. In this work, the hit rate against the EHoS dataset reached its highest rate of 65–70% during the standard and erase (*R*_*th*_ =400*v*_*ON*_) SAN simulations at high values of *U*_*avg*_. Other lifelong learning metrics such as Learning Curve Area (LCA) defined by Chaudhry et al. (2017), could be used to evaluate the SAN in future work to compare results.

For the dataset studied in this work, its sets had a wide range of U-Factor values. In datasets where the U-Factors of the sets are much more commonly higher values, data will appear very uniform and have few distinct characteristics. In these cases of high uniformity, the erase behavior would be quite potent in removing the high amount of commonalities and making the memories more distinct from one another. To ensure those similar traits are erased and forgotten, define the *R*_*th*_ value as an intermediate or high value. In fact, if the number of times the commonalities occur through the dataset is known prior, *R*_*th*_ can be calculated by the equation,


(8) $$R_{th}=I_{tot}f_{sim}v_{ON}$$

where *f*_*sim*_ represents the similarity frequency. For example, if there was a feature within one of the sets of the dataset that was highly common with a *f*_*sim*_ = 0.6 (i.e., 60%), and there were a total of 100 memories in the dataset, *R*_*th*_ could be set to a value of 60*v*_*ON*_ to erase the commonalities once all of them occurred. If the commonalities were desired to be erased sooner, *R*_*th*_ could be lowered. However, it should not be lowered to a point where it might remove other features deemed important to memories that might be more unique.

In terms of the other factors for the forgetting and predicting behaviors, *D*_*rate*_ and *P*_*th*_ have their own quirks. *D*_*rate*_ effectively defines the SAN's “attention span” when utilizing the forgetting behavior. When using a *D*_*rate*_ of 1.5% in Figure 10, memories on average were becoming unrecallable after ~100 timesteps. If *D*_*rate*_ were decreased this 100 timestep attention span would increase. If it is desirable for a system to possess a very long attention span, very low rates for *D*_*rate*_ would be intuitively preferred. However, it is key to remember too long of an attention span could lead to capacity saturation within the network which could then lead to catastrophic forgetting. *D*_*rate*_ should be tuned to a point where it is preventing capacity saturation, but also remembering things long enough for them to be useful. This attention span is very dataset and application specific, however it is reliable to say that datasets with low U-Factor sets will have a difficult time with any intermediate or high value for *D*_*rate*_. Since a low U-Factor set possesses more unique features, each feature typically occurs less often. A drop in occurrence rates means that the network has fewer opportunities to keep the memory reinforced *via* similar feature pairs. Datasets with at least some sets with high U-Factors would fare better with the forgetting behavior due to less unique features still giving opportunities to update some feature pairs more often. If one of the features in those pairs is common while another unique, the unique fingerprint of that memory can persist within the SAN and be recallable.

In terms of the threshold, *P*_*th*_, for the predicting behavior, higher values appear to be safest. Even at higher values however, the behavior appeared risky to utilize and often caused detrimental effects to the system's recall capability *via* capacity saturation. This behavior might need more adjustment to become safer to use. However, if one does wish to use the predicting behavior in a model, it is recommended to use a very high value [e.g., (*s*-1)*v*_*ON*_ where *s* is the number of sets within the dataset] for *P*_*th*_ regardless of the dataset.

## Conclusion

The simulations conducted in this work have demonstrated a segmented attractor network operating on the European Heads of State (EHoS) dataset. In these simulations, three metrics: hit rate, unique memory ratio, and recall occurrences, were used in measuring the network's lifelong learning success. In addition to the network's baseline, additional learning behaviors were studied to explore methods of enhancing the network's capability of association. These results show strong, lifelong learning capabilities of the segmented attractor network when used. Behaviors such as the erase and forgetting behaviors both showed capabilities that allowed the network's internal state to be curated over time to maintain relevant information and keep the network open to changes in its environment. The targeted end goal for this network would be to implement it in a specialized hardware for low-power, edge computing utilizing components such as memristors where computation can be performed in a fast, parallel process. Further work could be done on the network by evaluating it on other datasets of varied composure or exploring more advanced learning behaviors.

## Data availability statement

The datasets presented in this study can be found in online repositories. The names of the repository/repositories and accession number(s) can be found at: https://github.com/jones2a5/SAN_on_EHoS Located in the data folder.

## Author contributions

AJ acted as first author in this work by conducting the primary research and manuscript creation. RJ acted as the last author in this work by guiding the overall direction of the work, framing its outline, putting its results into meaningful context, and manuscript editing. Both authors contributed to the article and approved the submitted version.

## Funding

This work was initially submitted for review on Apr. 2022. This work was supported by the National Science Foundation under award numbers CCF-1718428 and ECCS 1926465.

## Conflict of interest

The authors declare that the research was conducted in the absence of any commercial or financial relationships that could be construed as a potential conflict of interest.

## Publisher's note

All claims expressed in this article are solely those of the authors and do not necessarily represent those of their affiliated organizations, or those of the publisher, the editors and the reviewers. Any product that may be evaluated in this article, or claim that may be made by its manufacturer, is not guaranteed or endorsed by the publisher.
