# Supplementary material for: Exploring the associative learning capabilities of the segmented attractor network for lifelong learning
Source: Front Artif Intell. 2022 Aug 1;5:910407. doi: 10.3389/frai.2022.910407 (PMC9376266; doi:10.3389/frai.2022.910407)
Supplement: Supplementary file 1 [file Data_Sheet_1.DOCX]

Supplementary Material

# Hit Rate, Unique Memory Ratio of Predictive Behavior
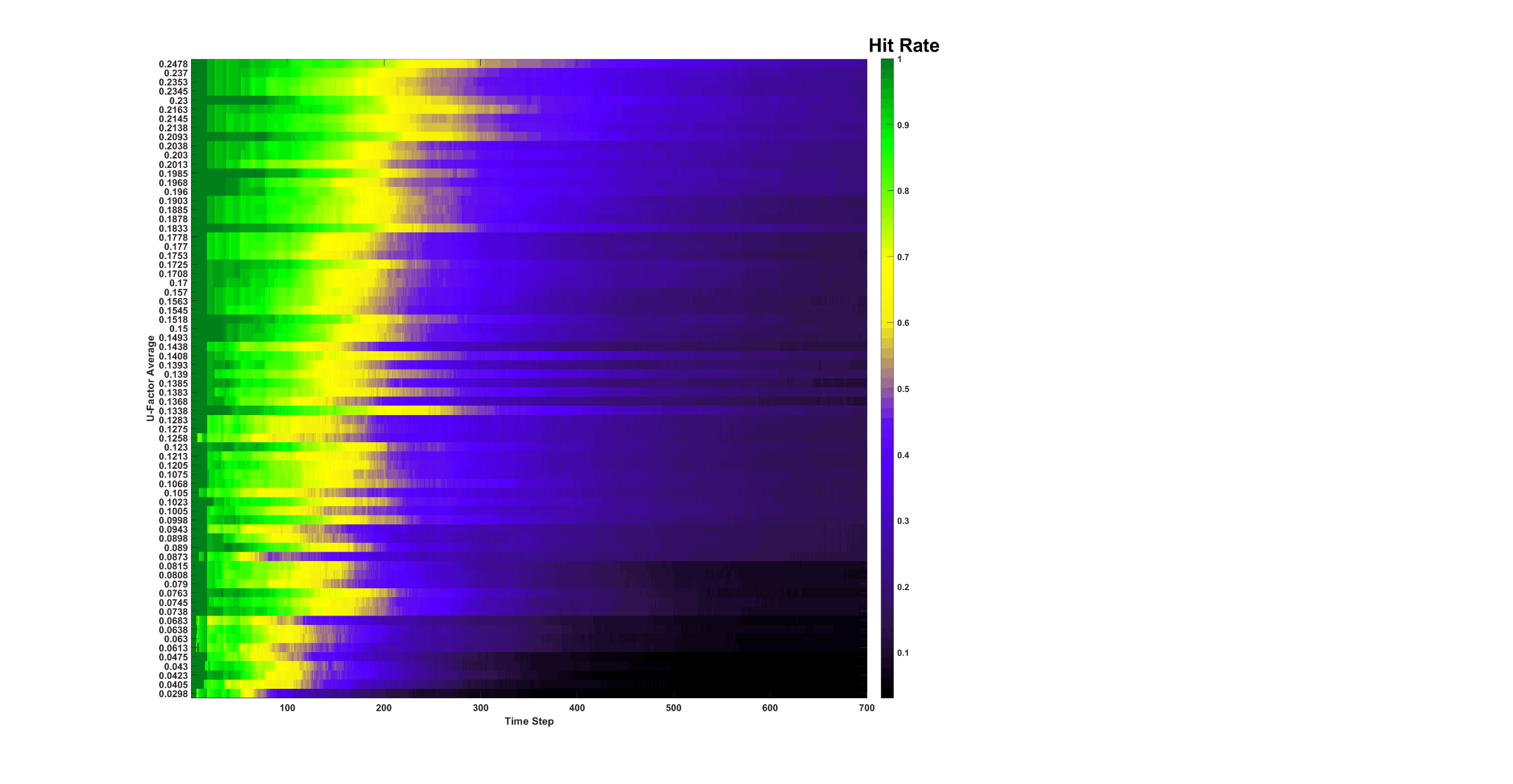
Supplementary Figure 1. Hit rate for the predictive behavior when *P_th_*=6*w_ON_*.


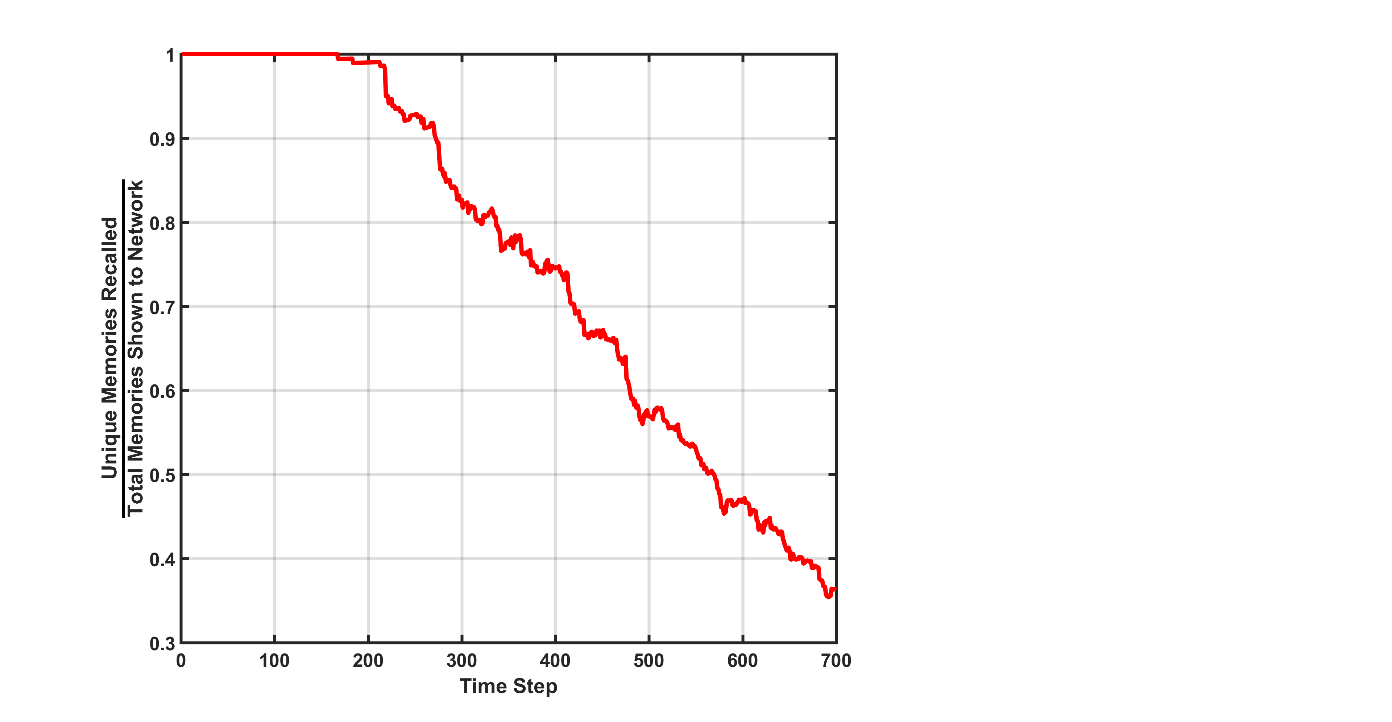


Supplementary Figure 2. Unique memory ratio for the predictive behavior when *P_th_*=6*w_ON_*.

# Recall Occurrences for Erase Behavior
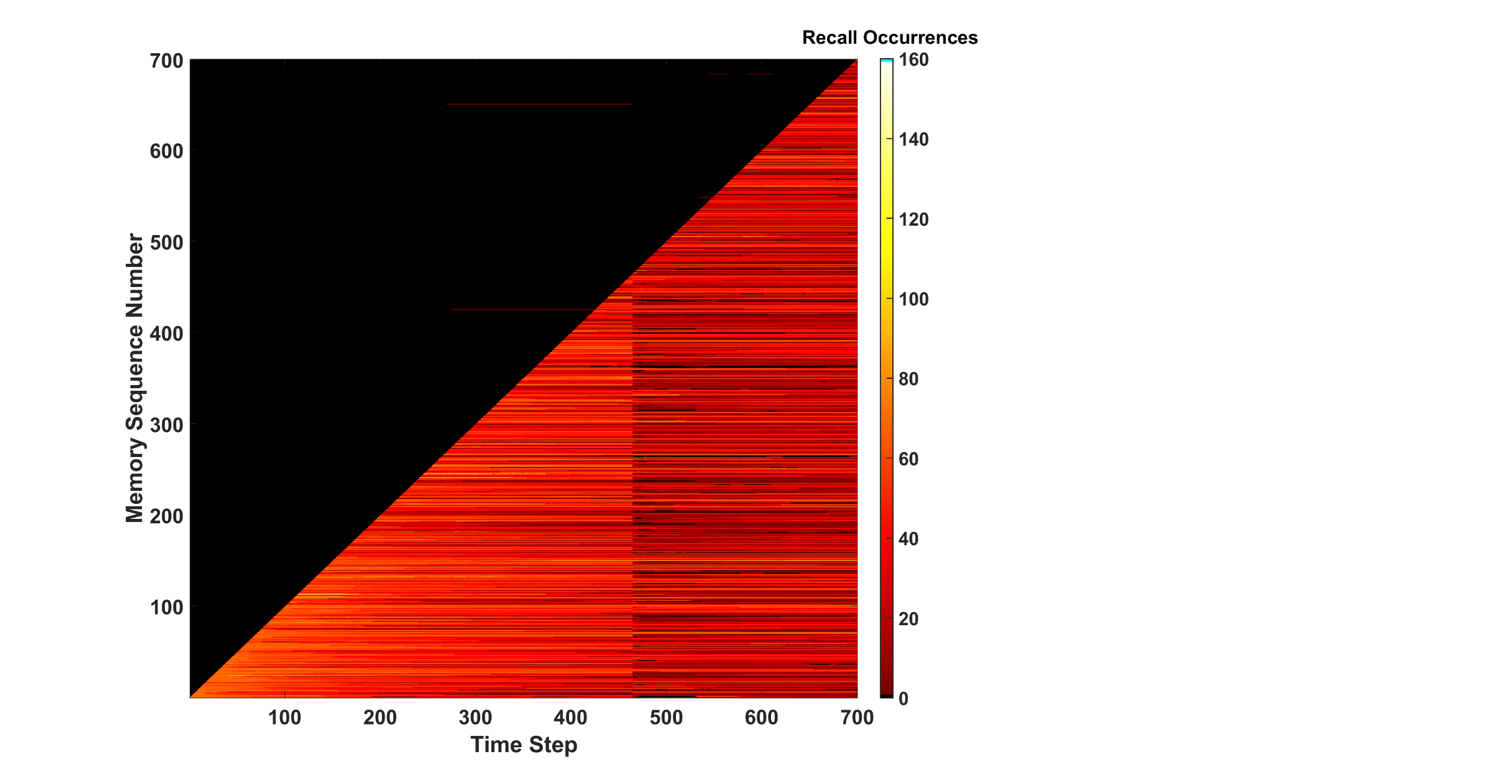
 Supplementary Figure 3. Recall occurrences heatmap for the erase behavior when *E_th_*=400*w_ON_*.


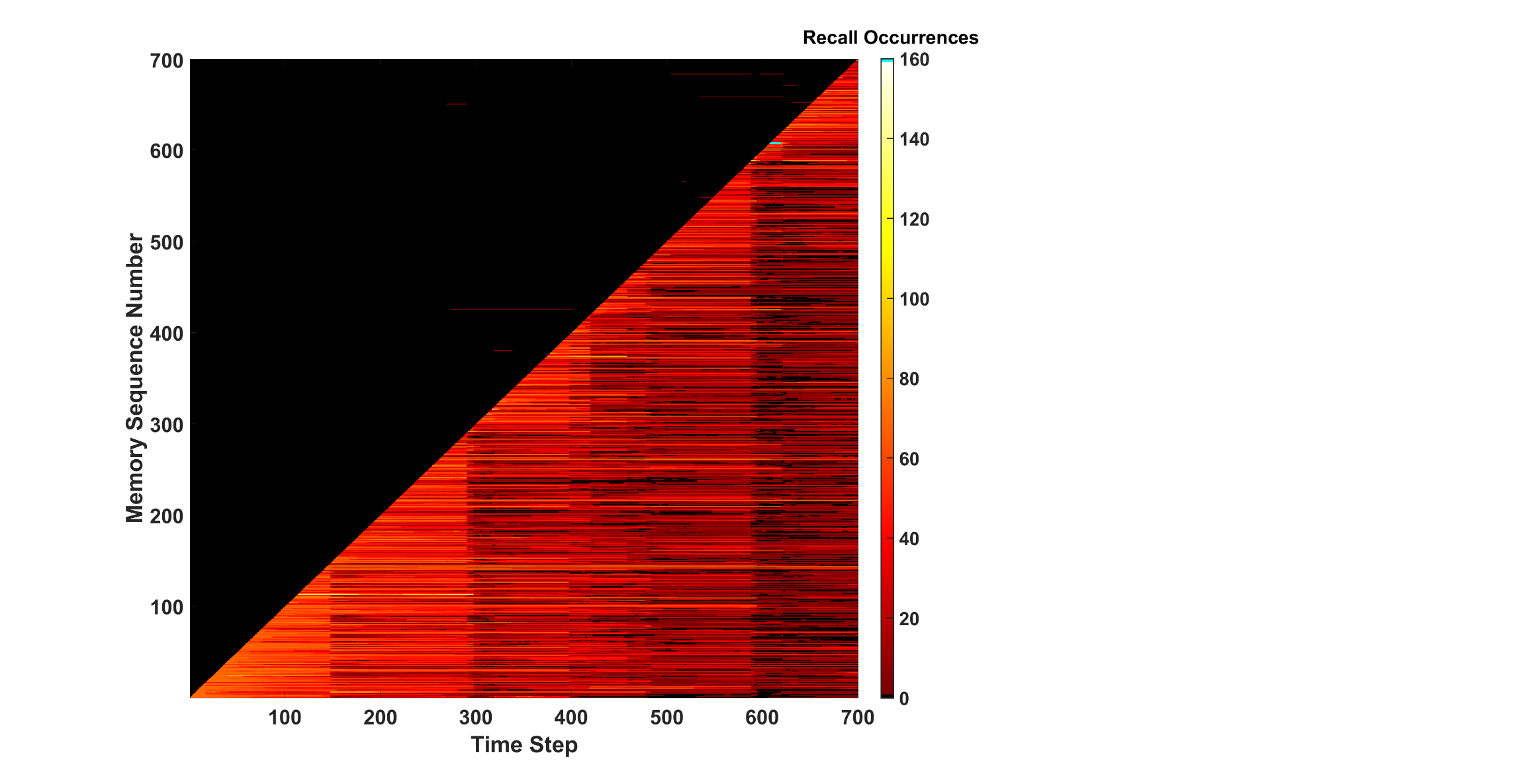


Supplementary Figure 4. Recall occurrences heatmap for the erase behavior when *E_th_*=200*w_ON_*.

# Hit Rate, Unique Memory Ratio, of Forgetting Behavior


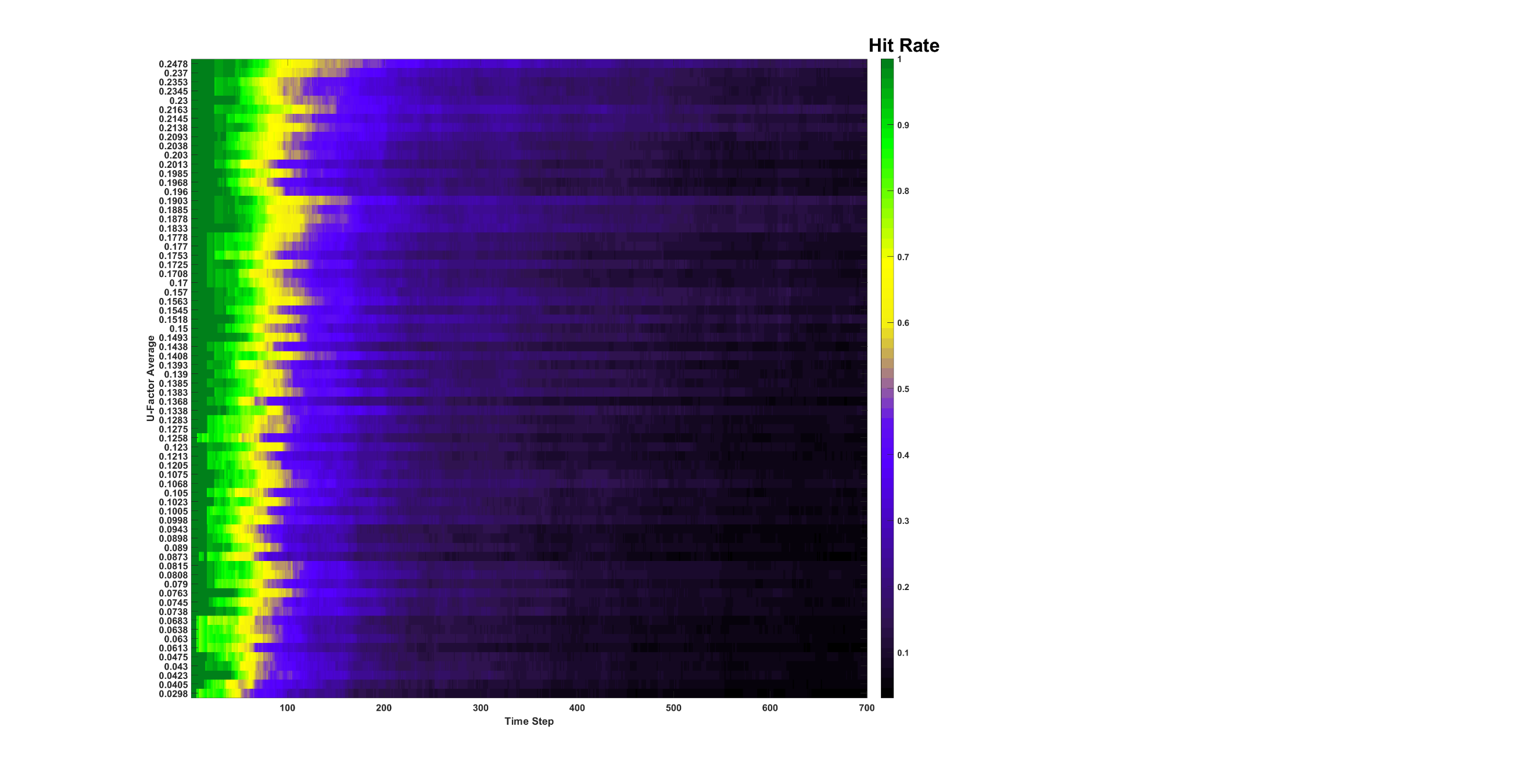


Supplementary Figure 5. Hit rate for the forgetting behavior when *r_stp_*=0.015.


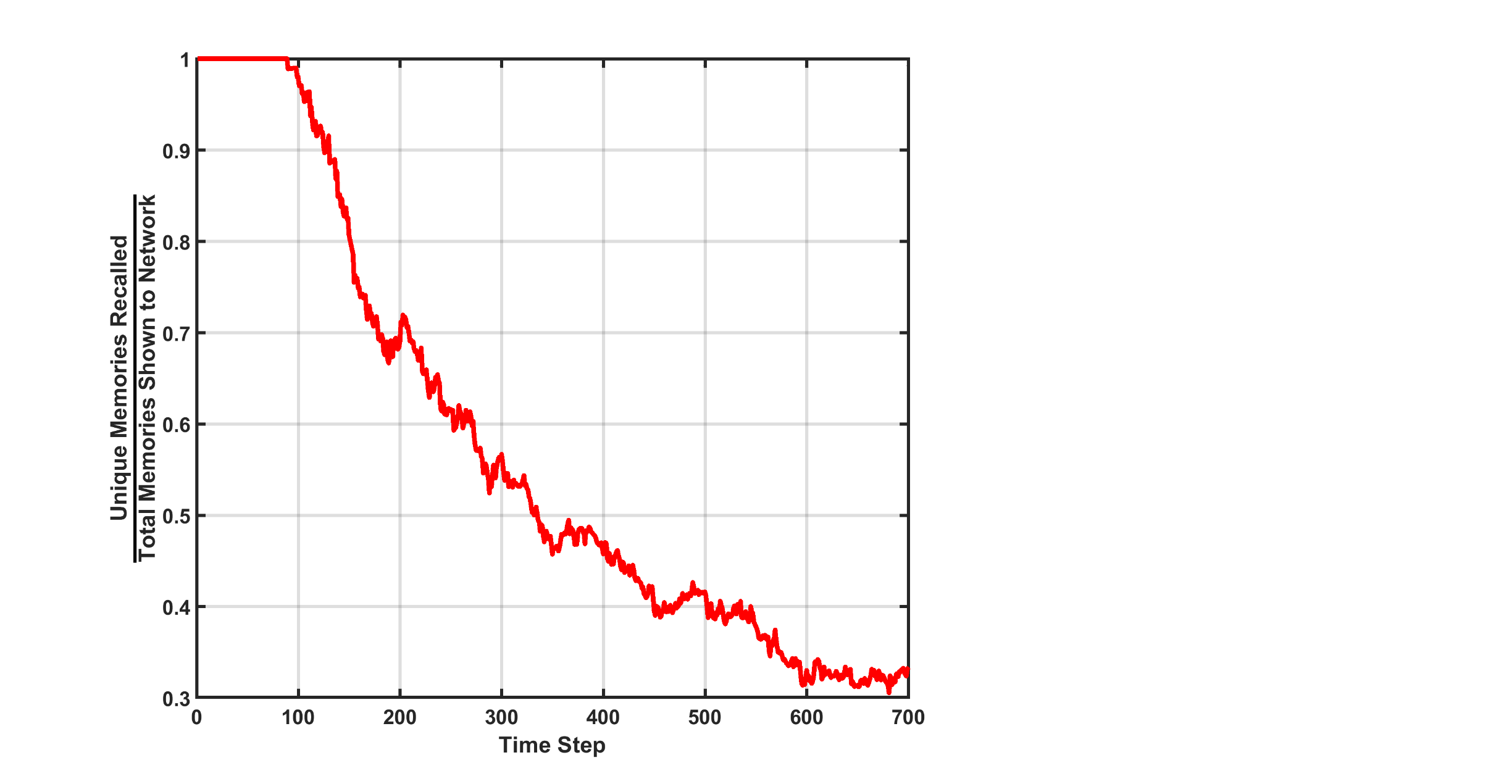


Supplementary Figure 6. Unique memory ratio for the forgetting behavior when *r_stp_*=0.015.

# Results of Behavior Ensemble with No Predictive Behavior


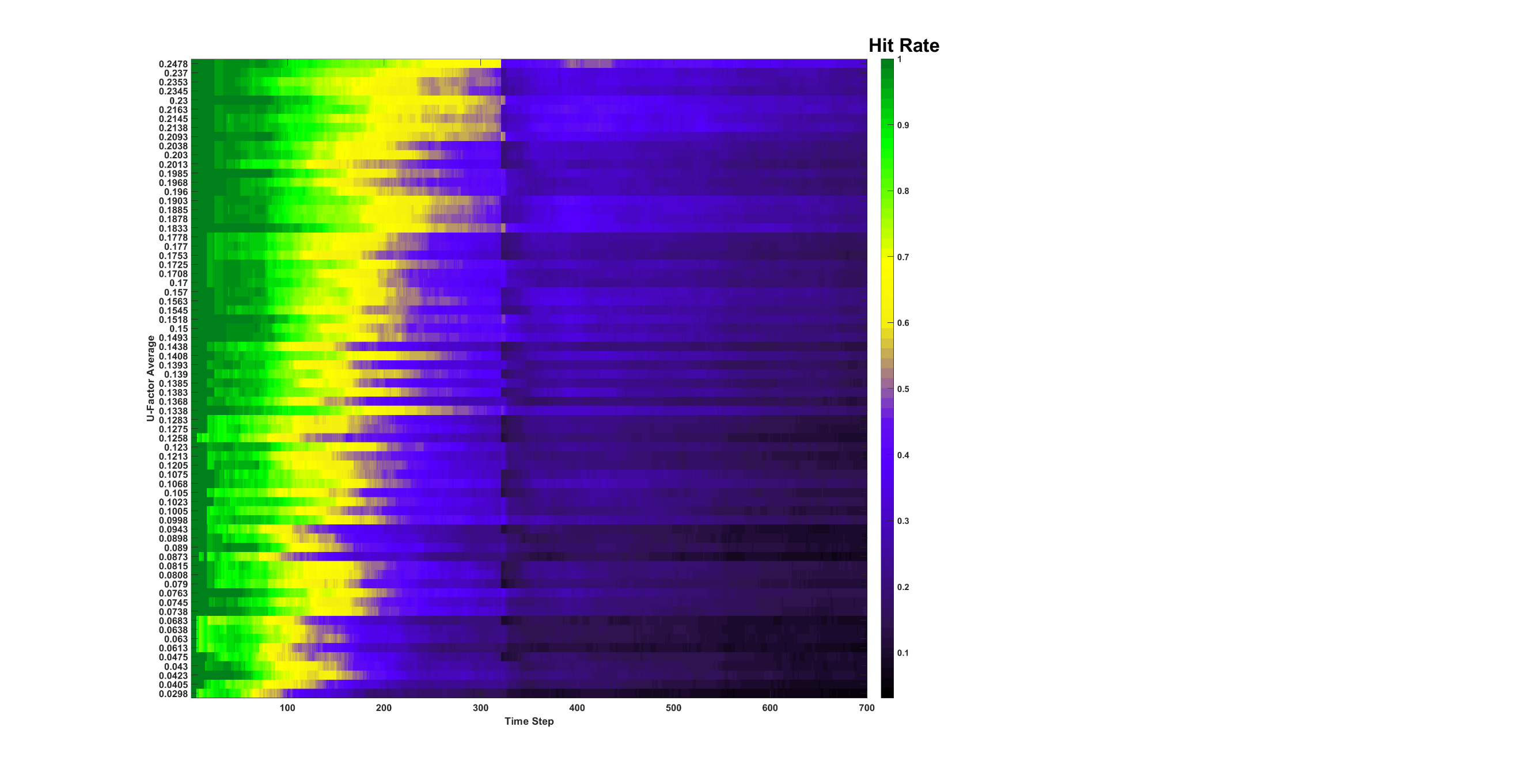


Supplementary Figure 7. Hit rate for the behavior ensemble when *E_th_*=200*w_ON_*, *r_stp_*=0.005. No predictive behavior was used and a similar result to Figure 11 was given.


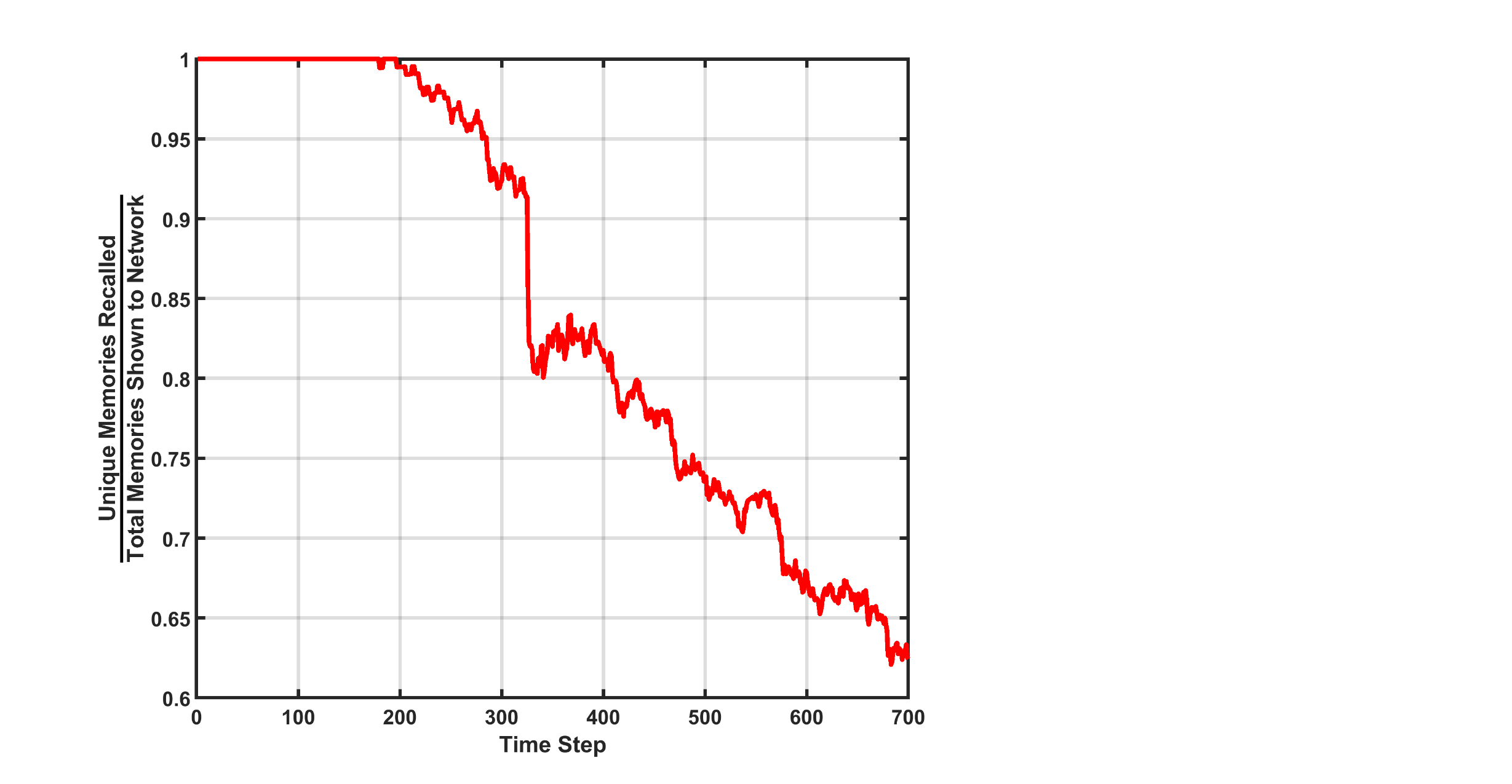


Supplementary Figure 8. Unique memory ratio for the behavior ensemble when E_th_=200w_ON_, *r_stp_*=0.005. No predictive behavior was used and a similar result to Figure 12 was given.


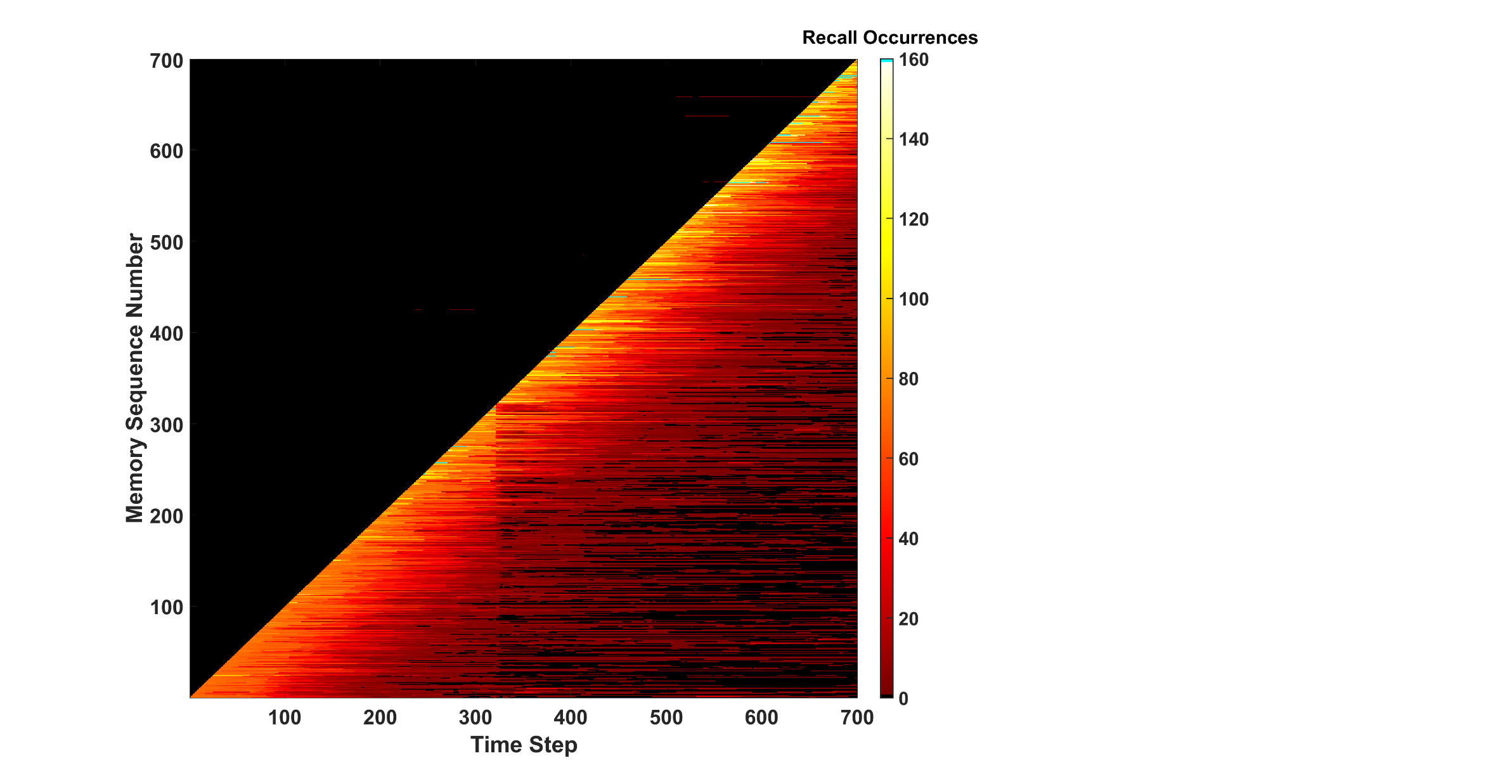


Supplementary Figure 9. Recall occurrences heatmap for the behavior ensemble when *E_th_*=200*w_ON_*, *r_stp_*=0.005. No predictive behavior was used and a similar result to Figure 13 was given.
